# Triple-Function-Intensified Photoelectric Immunosensing Platform for One-Step Detection of Cardiac Troponin I

**DOI:** 10.1021/acsami.5c06443

**Published:** 2025-08-21

**Authors:** Shin-Chwen Yeh, Wen-Shan Chen, Wen-Yin Ko, Kuan-Jiuh Lin

**Affiliations:** Department of Chemistry, 34916 National Chung Hsing University, Taichung 402, Taiwan

**Keywords:** nanoplasmonic, metal/semiconductor hybrids, photoelectric immunosensor, label-free, bioadhesive film

## Abstract

Integrating semiconductor nanowires with a nanoplasmonic metal surface substrate enables a real-time photocurrent response, offering a promising immunosensing platform. However, achieving one-step biorecognition detection of cardiac troponin I (cTnI) biomarker remains a challenge. Herein, we present the development of an antenna-engaged nanowire-inspired porous heterostructure comprising polydopamine-functionalized titanium dioxide nanowires integrated with an Au plasmonic layer (PDA/TNW/Au-PL) for ultrasensitive immunosensors under homemade white light-emitting diodes (LEDs). This deliberate combination of Au-PL, TNWs, and PDA-based trilayer hybrid electrode design adopts the following unique triple-function signal intensification characteristics: (i) interfacial engineering of type II heterojunction and Schottky junction synergistically improve the separation efficiency of electron–hole pairs, thereby significantly improving the photoelectric performance; (ii) strong absorbance across the entire visible range spectrum (400–800 nm) facilitates exceptional plasmonic resonance, enhancing light-harvesting efficiency; and (iii) the nanowire-inspired PDA layer-based nanocavities not only enhance the mobility of charge carriers but also enable the rapid, direct binding of the cTnI antibody onto the PDA surface within 10 min. Therefore, a one-step immunosensing protocol for label-free cTnI detection with a detection time of approximately 30 min is achieved without the requirement of secondary antibodies, amplification labels, or redox mediators. It demonstrates a wide linear sensing range from 0.001 to 1000 ng mL^–1^, with an ultralow detection limit of 0.001 ng mL^–1^. Our research provides a strong impetus for point-of-care diagnostics, enabling immediate and accurate therapeutic decision-making.

## Graphical abstract

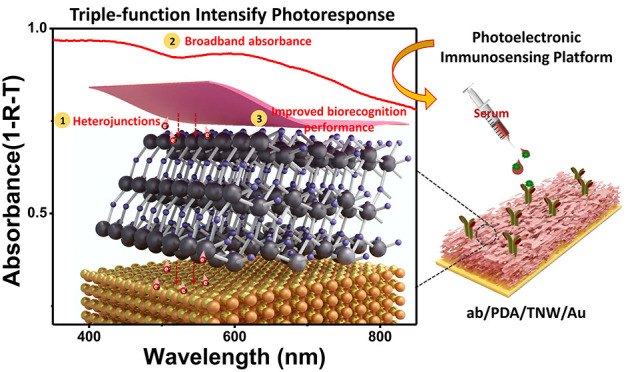

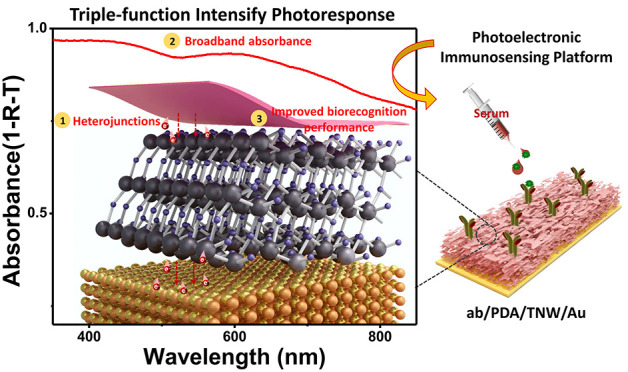

## 1 Introduction

Acute myocardial infarction (AMI), the most common cardiovascular disease, is recognized as a serious condition associated with a high mortality rate and is declared as the main cause of death by the World Health Organization (WHO) with 23.3 million global deaths by the end of 2030.^1,2^ Therefore, researchers aim to fulfill the major unmet need for early and accurate diagnosis, as well as preclinical research for AMI diseases. Cardiac troponin I (cTnI), which is released only in response to cardiomyocytes, is widely recognized as the gold standard biomarker for the early diagnosis of AMI and further clinical treatments. In healthy individuals, the normal blood concentrations of cTnI is between 1 and 50 ng L^–1^.^3,4^ Conventional analytical techniques for cTnI detection are mainly electrochemiluminescence (ECL) and enzyme-linked immunosorbent assay (ELISA).^5,6^ However, the multistep sample processing, long diagnostic time, and requirement of a well-trained operators as well as basic infrastructures are serious drawbacks of these approaches, which would highly limit the early detection of myocardial injury in patients. Compared with these detection methods, an immunosensing platform based on the gating effect of the antigen–antibody binding events has recently received intensive attention as a powerful diagnostic platform for cTnI detection owing to its capability for ultrasensitive, selective, and real-time sensing.^7–9^ In addition, the easy integration with signal processing circuits and low cost make it possible for point-of-care testing (PoCT) devices to offer added value in facilitating on-site diagnosis for patients. The coupling of immunochemical reactions with different sensing strategies, including electrochemical, photoelectric, fluorescent, luminescent, and nanoplasmonic sensors, has been developed and successfully employed for the analysis of cTnI proteins with high sensitivity.^10–13^ In the past decade, the detection limit of cTnI has been reported to be 0.001 ng/mL using the nanoplasmonic immunoassays.^14^ However, multiple laboratory steps and reliance on complex signal amplification components are still required, making them unsuitable for PoCT device applications.^2^

Recently, nanoplasmonic hybrid semiconductor/metal-based photoelectrodes have been recognized as promising candidates for the photoelectric applications due to their fast and remarkable photocurrent response, attributed to the promising plasmon-induced charge separation effect,^15–18^ which also provides conditions for the construction of photoelectric immunosensors as a powerful real-time diagnostic platform for the use in PoCT technologies.^19,20^ The combination of Au nanostructures, which exhibit collective oscillations of conduction electrons at visible light frequencies, with semiconductors has been confirmed to influence the distribution of electromagnetic energy.^21,22^ This, in turn, can significantly alter the photoelectric response under visible light irradiation, beneficial for performing clinical laboratory testing at the patient’s site.^23^ Nevertheless, the construction of the corresponding sensor configuration involves multiple incubation and washing steps because of the complex amplification steps, rendering it unsuitable for the PoCT system. Recently, our group successfully designed a visible-light-driven and label-free photoelectric immunosensing platform based on two-dimensional (2D) porous nanoheterostructures composed of Au nanoparticles and TiO_2_ nanowires, which exhibited one-step sensitive detection of α-fetoprotein (AFP) and immunoglobulin G (IgG) biomarkers without amplification strategies such as the use of labeling materials, blocking reagents, and linker molecules (Figure S1).^24,25^ However, this method is not applicable to small biomolecules such as cTnI targets, as the surface loading accommodated by cTnI antibodies is limited by physical immobilization restrictions, despite the large surface area and high porosity of TiO_2_ nanowires. It has been observed that the antibodies are immobilized on the surface of the substrate through noncovalent interactions, including hydrophobic forces, van der Waals forces, π–π interactions, and electrostatic interactions.^26,27^ These interactions are relatively weak and reversible, leading to potential antibody desorption in the presence of competing proteins and resulting in inefficient antigen binding, thereby reducing the antigen recognition ability and compromising the sensor performance.^28^ Therefore, to improve stability and binding efficiency, covalent immobilization strategies are necessary to achieve higher sensitivity and accuracy in immunosensing detection.^29,30^

Polydopamine (PDA), an environment-friendly semiconducting biopolymerized material with extremely strong adhesive property and excellent biocompatibility caused by its diverse functional groups, like catechol, amine, and imine, has been widely used as a binding agent to form thin bioadhesive films for antibody immobilization or further bioconjugation.^31–38^ PDA-based materials have recently piqued the interest of researchers for electrochemical-related applications due to their promising electrochemical properties, such as enhanced conductivity, good electron transfer kinetics, and better electrochemical activity from their redox catechol groups.^39,40^ Besides, PDA has been proven to exhibit strong visible light absorption and an outstanding ability to generate photocatalytic heterojunctions with plasmonic and transition metal oxides, making it a potential prospect for photoelectrodes.^41–43^ Thus, PDA might be a suitable candidate for modifying our hybrid semiconductor/metal nanostructures to improve the performance of small biomolecular target detection when used as a photoelectric immunosensor, in which PDA serves both as an adhesive interlayer for the effective immobilization of cTnI antibody and as a heterojunction-promoting functional coating for enhancing the efficiency of photoelectric conversion by creating a synergistic effect between the heterojunction and Schottky junction.^44^ In addition, there is no report so far on the utilization of the PDA-functionalized Au/TiO_2_ nanocomposite to design a photoelectric immunosensor for the detection of cTnI, which is worthy of investigation.

In this study, a simple yet effective and specific constructive photoelectric immunosensing platform with a one-step assembly process for the detection of cTnI is developed based on the coating of a PDA bioadhesive nanomembrane on our designed titanium nanowires/2D-arrayed Au-nanoparticle plasmonic layer (PDA/TNW/Au-PL) to create a novel nanoplasmonic hybrid trilayer photoelectrode. The PDA nanomembrane facilitates the use of only a single analyte-specific antibody without the need for other additional cross-linkers or signal amplification reagents for the one-step construction of a label-free immunosensor within just 10 min. Under optimal conditions, it achieved a wide detection range of 0.001–1000 ng mL^–1^, a low detection limit of 0.001 ng mL^–1^, and excellent selectivity under visible light illumination. The design’s simplicity, not only in terms of the easy to fabricate scalable nanostructured photoelectrodes but also the fast biosensing assay configuration, as well as the rapid detection response allows the potential applicability to quickly detect cTnI on-site in clinical settings, without the need of complex instruments or specialized personnel, thereby facilitating the early diagnosis of various cardiac diseases.

## 2 Results and Discussion

The detailed stepwise process of the PDA/TNW/Au-PL immunosensing platform for the detection of cTnI is depicted in Figures 1A and S2. First, a 2D array of Au-PL layers for enhanced nanoplasmonic resonance is fabricated on FTO using a sputtering deposition process, followed by microwave plasma heat treatment. Then, a cavity-rich nesting TiO_2_ nanowire (TNW) layer is grown on the surface of the Au-PL layers, undergoing a process of spontaneous autoxidation via a simple and facile alkaline thermal treatment, where the Schottky junction formed at the interface between the TWN layer and the Au-PL layer could enable effective nanoplasmonic coupling, resulting in enhanced visible light absorption properties. Finally, a biocompatible PDA nanofilm is coated on the TNW surface via a hierarchical self-assembly process in mild alkaline aqueous solutions to form the PDA/TNW/Au-PL photoelectrode. The PDA nanomembrane could covalently cross-link the cTnI antibody agents and amplify the photoresponse current of the photoelectric process owing to the formation of a type II heterojunction at the interface of PDA and TNW to effectively enhance interfacial interactions and thereby lead to superior dielectric properties.^45^ Afterward, a one-step rapid antibody array can be developed in 10 min by chemically immobilizing the cTnI antibody (denoted as anti-cTnI or ab) directly onto the PDA nanomembrane through the Michael addition and Schiff base reactions (Figure 1B),^46^ which can induce to reduce the desorption rate of anti-cTnI from the sensor surface, thereby enhancing the sensing sensitivity by increasing the number of stable antigen–antibody complexes formed on the surface, which in turn promotes a stronger detectable signal due to the greater availability of active binding sites. This process of PDA coating can address the challenges highlighted in previous studies related to the difficulties of strong antibody adsorption occurring through complex surface treatments such as the amine-modified method. Finally, a one-step protocol of an immunosensing platform based on the ab/PDA/TNW/Au-PL platform is used for the photoelectric detection of cTnI antigen biomarker under the irradiation of homemade white light-emitting diodes (LEDs). Binding of cTnI to its specific antibody, enabled through engineered biological recognition, would lead to increased interfacial resistance and decreased photocurrent on the PDA/TNW/Au-PL due to the curbing of electron transfer by nonelectroactive proteins.^47^ Therefore, cTnI can be selectively, sensitively, and quantitatively detected by measuring the photocurrent response change occurring on the PDA/TNW/Au-PL.

**Figure 1:**
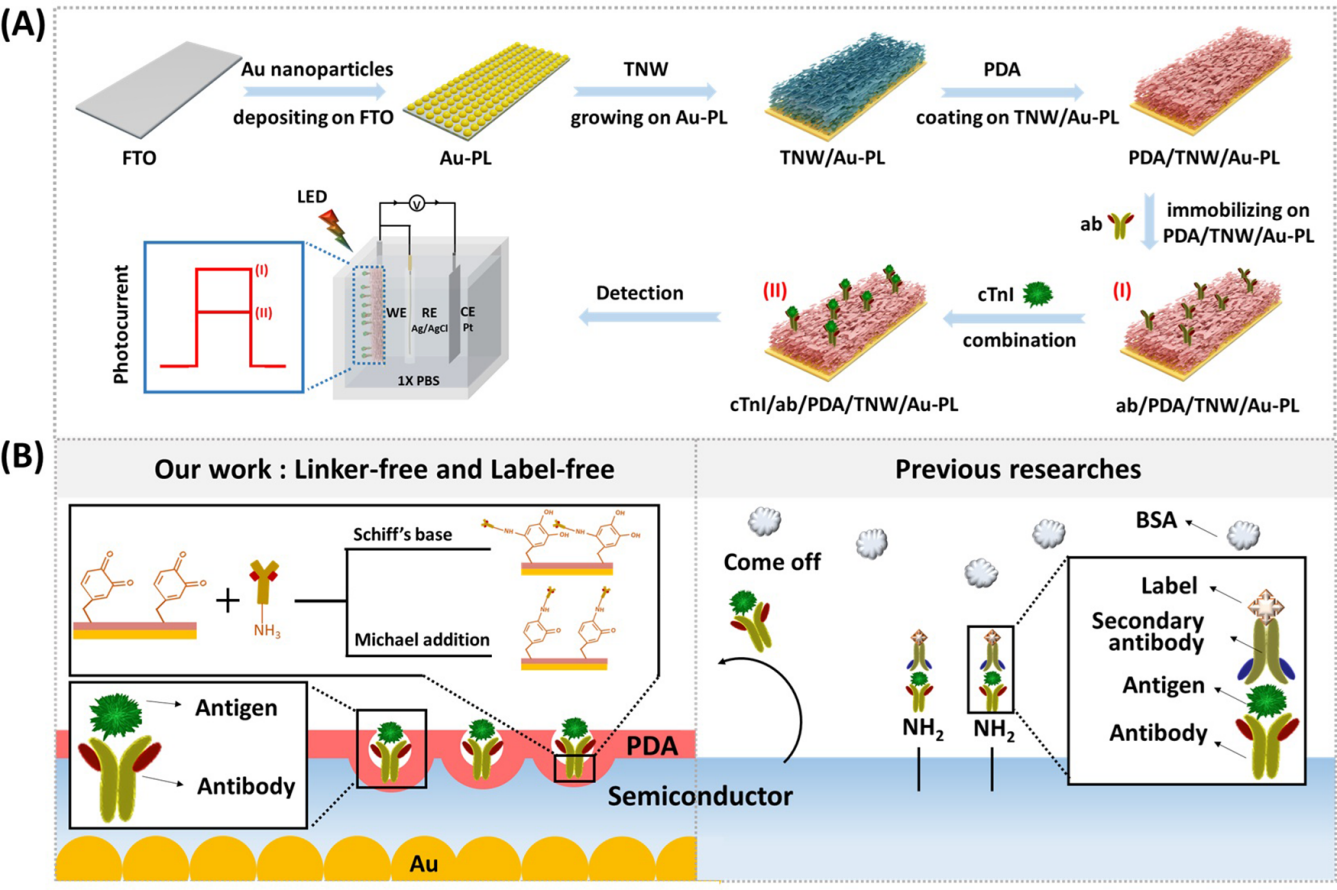
(A) Schematic illustration of the fabrication process of the nanoplasmonic hybrid trilayer (PDA/TNW/Au-PL) photoelectrode and the assembly of the immunosensing platform for cTnI detection. (B) Schematic illustration of the proposed linker-free and label-free immunosensing detection.

Figure 2 shows the morphology and structural characterization of the as-prepared PDA/TNW/Au-PL photoelectrode. The surface morphologies of the respective layers from bottom to top of PDA/TNW/Au-PL are exhibited in Figure 2A–C. The bottom layer of Au-PL, deposited onto the surface of the FTO substrate, is a 2D nanoplasmonic layer comprising densely packed Au nanoparticles, which exhibits excellent plasmonic optical behavior (Figure 2A). When viewed from the top of TNW/Au-PL, as shown in Figure 2B, the middle layer of TNW deposited on the Au-PL layer represents a pore-rich and cavity-rich nanoframework constructed from TiO_2_ nanowires. After coating the top layer of PDA onto the TNW surface (Figure 2C), there is no noticeable change in the TNW porous nanoframework, indicating that the mild alkaline conditions in the preparation of PDA are evolving for modifying only the surface structure and thus are beneficial for preserving the integrity of the TiO_2_ wire-like array geometry. In our experiment, dopamine spontaneously polymerized into PDA, and was strongly anchored to the Ti atoms of TiO_2_ due to the strong metal coordination ability of the catechol groups in PDA,^48^ making PDA an excellent bioadhesive membrane for improving the binding affinity between TNW and cTnI antibodies (Figure 2E). The coexistence of PDA and TiO_2_ in the sample is further confirmed by the Fourier-transform infrared (FTIR) spectrum shown in Figure S3. In the TNW/Au-PL spectrum, the three peaks located at <600, 1601, and 3310 cm^–1^ can be attributed to the stretching vibrations of Ti–O–Ti in the TiO_2_ lattice, bending vibration of Ti–OH groups, and the asymmetrical and symmetrical stretching vibrations of the hydroxyl group (−OH) from physically adsorbed water on TiO_2_.^48^ Besides, the sharp peak at 2360 cm^–1^ corresponds to the stretching vibration of TiOO–H.^49^ After coating PDA on TNW, two new broad peaks appear, in which the peak at 1055 cm^–1^ is attributed to the C–N stretching and the broadband located around 1800–1520 cm^–1^ is related to C=O, C=C, and the bending vibration mode of N–H bonding due to catechol groups of dopamine at the surface.^50–52^ These results support the fact that the TNW/Au-PL modified by PDA with our process is successful.

**Figure 2:**
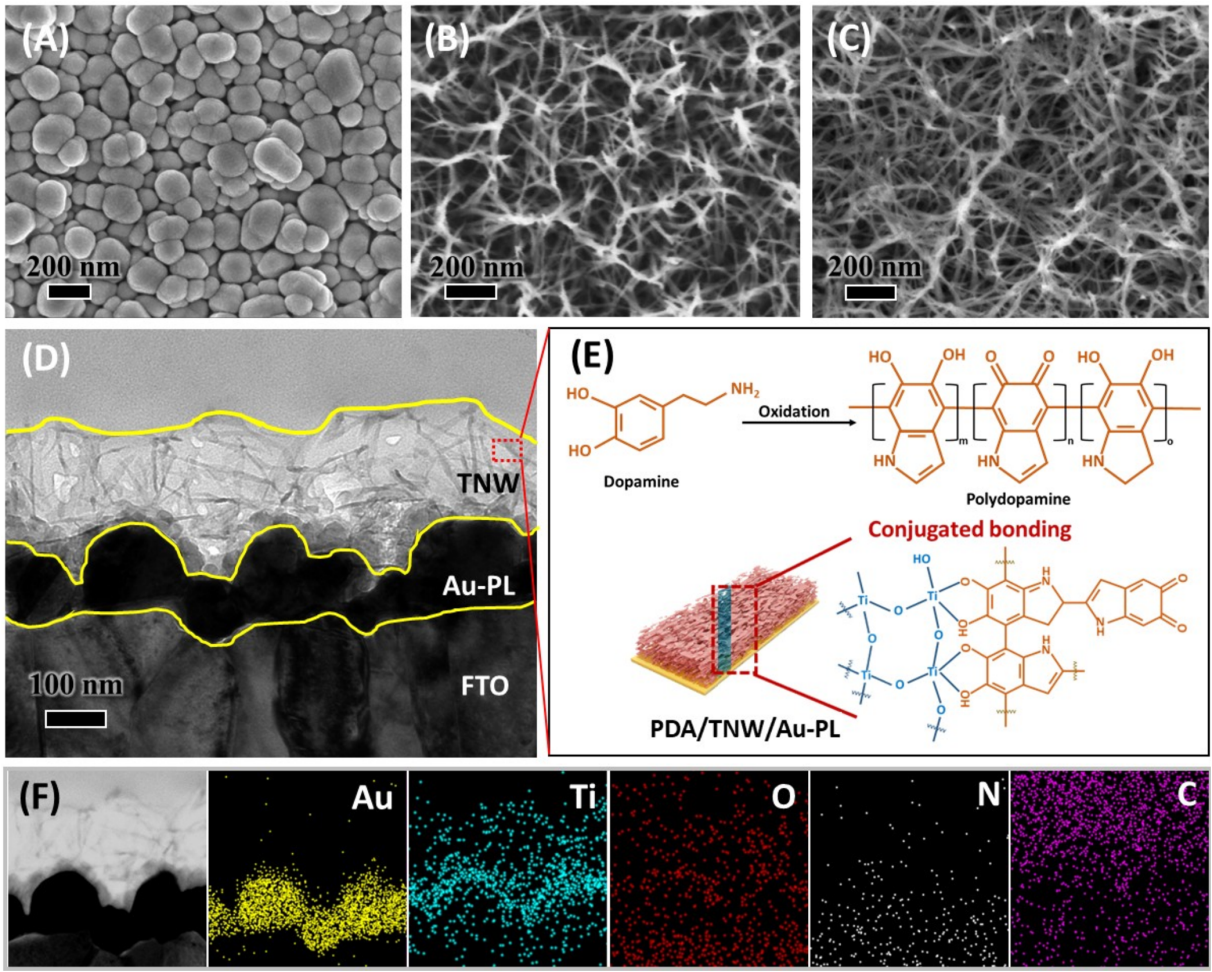
Morphological and structural characterization of PDA/TNW/Au-PL. SEM images of the surface morphologies of different layers: (A) Au-PL deposited on FTO, (B) TNW layer grown on the Au-PL surface, and (C) PDA coated on the TNW/Au-PL surface. (D) Cross-sectional TEM images of PDA/TNW/Au-PL. (E) Schematic representation of the formation of conjugated bonding between TNW and PDA. (F) EDS-mapping images of PDA/TNW/Au-PL for Au, Ti, O, N, and C elements.

To further observe the structure of PDA/TNW/Au-PL, TEM cross-sectional analysis is performed (shown in Figure 2D). It is noted here that the porous TNW with a thickness in the range of 150–200 nm on the Au-PL layer can be observed, and its rich pores and cavities built from free-standing TiO_2_ nanowires can provide ample sites for adsorbate molecules to contact because of the increased surface area. The results also confirm that the bioadhesive membrane of PDA is uniformly and thinly coated on the TNW surface without destroying the nanowired structure or blocking the cavities between nanowires, as the porous TNW structure remains unchanged even after the PDA coating process, consistent with the results shown in the SEM characterizations. In addition, the energy-dispersive spectrometry (EDS) elemental mapping images of PDA/TNW/Au-PL reveal that the granules at the bottom are identified as metallic Au, and the area above is rich in Ti and O contained in TiO_2_, indicating the successful formation of TNW/Au-PL (Figure 2F). Moreover, the N and C elements of PDA are evenly distributed across the TiO_2_ nanowires, which proves that the PDA coating is successfully formed on the surface of the TNWs.

A key step in improving the photocurrent of photoelectric immunosensors for practical use is to increase the optical absorption in the visible range. The absorption spectra of Au-PL, TNW, TNW/Au-PL, and PDA/TNW/Au-PL are also determined using *A* = 1−*R*–*T*, where *R* and *T* are the normalized reflection and transmission, respectively, as shown in Figures 3A and S4. As seen from the spectra, a poor absorbance is observed in the visible region for the TNW sample, which is ascribed to the wide optical band gap of TiO_2_. The Au-PL layer exhibits electroplasmon characteristics with a broad visible wavelength light absorption spectrum (Figure 3A). This absorption behavior primarily benefits from the multiple plasmon resonances of the deposited Au nanoparticles with a wide size distribution, along with the strong plasmon near-field coupling between nanoparticles located close to each other.^24,53,54^ The TNW/Au-PL hybrid bilayer shows an obvious enhancement of absorption and exhibits remarkable broadband absorption over the entire visible range spectrum. Particularly, the absorption spectrum is flat over the whole range of 400–800 nm, with a minimum absorbance of 0.66 and a maximum absorbance of 0.96. The absorption enhancement arises from the combined effects of interference-induced antireflection and the excitation of local photonic modes at the interface of TiO_2_ and Au.^55^ Besides, the pore-rich and cavity-rich TNW layer could effectively trap the light by forcing multiple internal light scattering to effectively extend the optical path length, thereby reducing optical reflection and then enhancing light absorption. (Figure S4) The PDA/TNW/Au-PL hybrid trilayer also features a broadband optical absorption range similar to that of the TNW/Au-PL one, but with a slight increase in absorption intensity.

**Figure 3:**
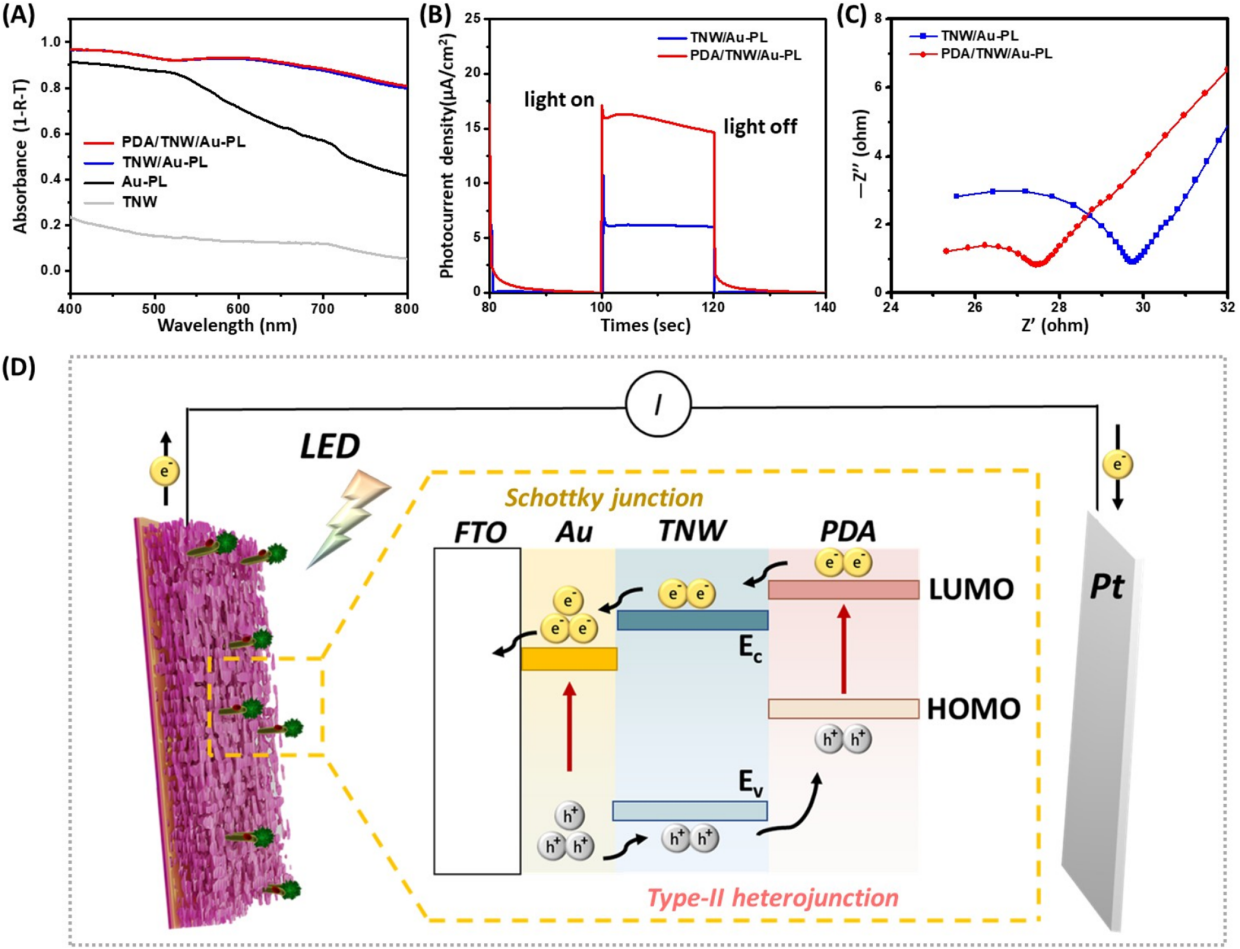
(A) UV–visible spectra of Au-PL, TNW, TNW/Au-PL, and PDA/TNW/Au-PL. (B) Photocurrent response of the TNW/Au-PL and PDA/TNW/Au-PL photoelectrodes measured in 1× PBS buffer solution under homemade white light LED illumination. (C) EIS Nyquist plot of TNW/Au-PL and PDA/TNW/Au-PL measured in a 0.5 mM [Fe­(CN)_6_]^3–/4–^ solution containing 0.1 M KCl in the frequency range of 0.01 and 1 MHz. (D) Schematic illustration of the photogenerated electron transfer mechanism of PDA/TNW/Au-PL.

To further understand the photoactive advantages of the PDA/TNW/Au-PL photoelectrode, the photocurrent of the electrode was measured in real time to evaluate their potential as photoelectric biosensors under visible light irradiation. For constructing a sensitive photoelectric sensor, high photoelectric activity is essential, which would correspond to the magnitude of the photocurrent. As displayed in Figure 3B, the photocurrent of PDA/TNW/Au-PL was 2.74 times higher than that of TNW/Au-PL in 1X PBS buffer solution under homemade white LED illumination. This significant improvement in the photocurrent is attributed to the introduction of the PDA coating, which forms a high-quality interface with the TNW surface. The presence of PDA not only improves light absorption but also facilitates the transfer of photogenerated electrons, thereby inhibiting the recombination of photoexcited electron–hole pairs and boosting photosensitivity. The photogenerated electrons in PDA would efficiently transfer from its conduction band to the conduction band of TiO_2_, owing to the type II band edge alignment and strong chemical bonding at the interface. As the DA molecules bond with TiO_2_, a new p-orbital is introduced, which allows the photogenerated electrons in PDA to be directly transferred from the edge of this new p-orbital into the conduction band of TiO_2_, creating a direct pathway for electron transfer.^56,57^ In addition, PDA can effectively promote the spatial separation of photogenerated charges (holes placed on dopamine) and electrons within TiO_2_ by transferring the photogenerated holes in TiO_2_ from its valence band to that of PDA, resulting in the inhibition of charge recombination.^58^ Furthermore, the PDA bioadhesive membrane features an extended π-conjugated electron system, which could provide lower charge-transfer resistance and higher charge carrier mobility (Figure 3C), which is beneficial for photocurrent enhancement. However, despite these advantages facilitated by PDA coating, a gradual decline in the photocurrent is still observed over time. This slight degradation is likely due to the photoinduced oxidation of PDA under continuous illumination, during which the catechol groups in PDA gradually oxidize to quinone structures, altering the electronic structure of PDA and diminishing its light-harvesting and charge-transfer capabilities.^56,59^ To mitigate this issue, strategies such as optimizing the PDA layer thickness or introducing a protective coating may help improve the photocurrent stability under light exposure of the sensor, thereby enabling the reliable validation of its sensing performance in real clinical samples.

A schematic mechanism for the generation, separation, and transfer of the charge carriers in the PDA/TNW/Au-PL photoelectrode is proposed, as depicted in Figure 3D.^58,60^ First, PDA would absorb photons with sufficient energy to induce a π–π* transition upon visible light irradiation, causing electrons in the excited state to move from the highest occupied molecular orbital (HOMO) of PDA to its lowest unoccupied molecular orbital (LUMO). Then, the photoexcited electrons in PDA would flow thermodynamically quicker to the conduction band of TiO_2_ due to the more negative conduction-band potential of TiO_2_. Also, PDA is a kind of p-type semiconductor that can enable rapid kinetic transfer at the interface with TiO_2_ and lead to quick transfer of its photogenerated holes to the valence band of TiO_2_, which is useful for carrier separation. Afterward, the electrons transferred to the conduction band of TiO_2_ further transferred to Au due to e^—^ migration through the Schottky barrier and finally injected into the FTO electrode. On the other hand, the 2D Au-nanoparticle layer can effectively utilize visible light because of its strong SPR effect, which plays a key role in enhancing the photoactive and photoelectrical properties. These results confirm the potential of the PDA/TNW/Au-PL photoelectrode as a photoelectric immunosensing platform for cTnI antigen detection.

Electrochemical impedance spectroscopy (EIS) is a powerful technique to monitor the changes in surface features and characterize interfacial properties related to the biorecognition and assembly processes on the electrode. The electrochemical impedance Nyquist plots of PDA/TNW/Au-PL after the modification of the cTnI antibody and cTnI antigen are shown in Figure 4A. Compared with the PDA/TNW/Au-PL photoelectrode, the semicircle diameter in the high frequency region increased after the electrode was immobilized with anti-cTnI, which demonstrated the increased charge-transfer resistance due to the lower electron transfer kinetics at the electrode surface resulting from the hindrance and insulation properties of anti-cTnI. The diameter further increased when the photoelectrode was modified with cTnI, implying successful specific recognition between anti-cTnI and cTnI. These results demonstrated that the proposed one-step ab/PDA/TNW/Au-PL photoelectric platform could be applied to determine cTnI. The photoelectron conversion of the photoelectrode is essential in achieving a photoelectric immunosensor. Thus, the construction steps of the photoelectric immunosensor could be verified by measuring the photocurrent response. Figure 4B illustrates the changes in photocurrent response of the designed sensor before and after the immobilization of the cTnI antibody and subsequent antigen binding. First, the photocurrent increased sharply and then reached a relatively steady value within approximately 3 s upon light-on across all measurements, indicating a fast sensing response for cTnI detection. As a result, the total detection time for cTnI was shortened to around 31 min, comprising a 30 min incubation period for antigen–antibody interaction and a few seconds for signal acquisition, achieving a competitive or shorter detection time than many existing photoelectrochemical, colorimetric, or fluorescence biosensors (Table S2). In addition, we observed that the photocurrent intensity decreased after the modification of anti-cTnI. The decrease in the photocurrent can be attributed to the immobilized biomolecule, which would reduce the electron transfer kinetics and partially hinder electron transfer from PDA to TiO_2_, leading to the recombination of photogenerated holes and electrons. The photocurrent intensity would further decrease after the biorecognition of cTnI with anti-cTnI. Therefore, the quantitative analysis of cTnI can be performed based on the measurement of the decrease in photocurrent intensity. To clarify the underlying mechanism responsible for the observed photoelectric signal variation, we attribute the photocurrent modulation to specific antigen–antibody interactions occurring at the PDA/TNW/Au-PL electrode interface. Upon white light illumination, the trilayer photoelectrode generates a strong photocurrent due to the enhanced light harvesting enabled by the Au plasmonic layer and TNW-induced optical scattering as well as the efficient charge separation and transport facilitated by a type II heterojunction at the PDA/TiO_2_ interface and Schottky junction at the TiO_2_/Au-PL interface. When anti-cTnI is immobilized on the PDA surface and subsequently captures cTnI antigens, a nonelectroactive layer is formed, resulting in a photocurrent decrease due to the increased interfacial resistance to block photogenerated electron transport. This signal suppression confirms the successful fabrication and functional operation of the photoelectric immunosensor.

**Figure 4:**
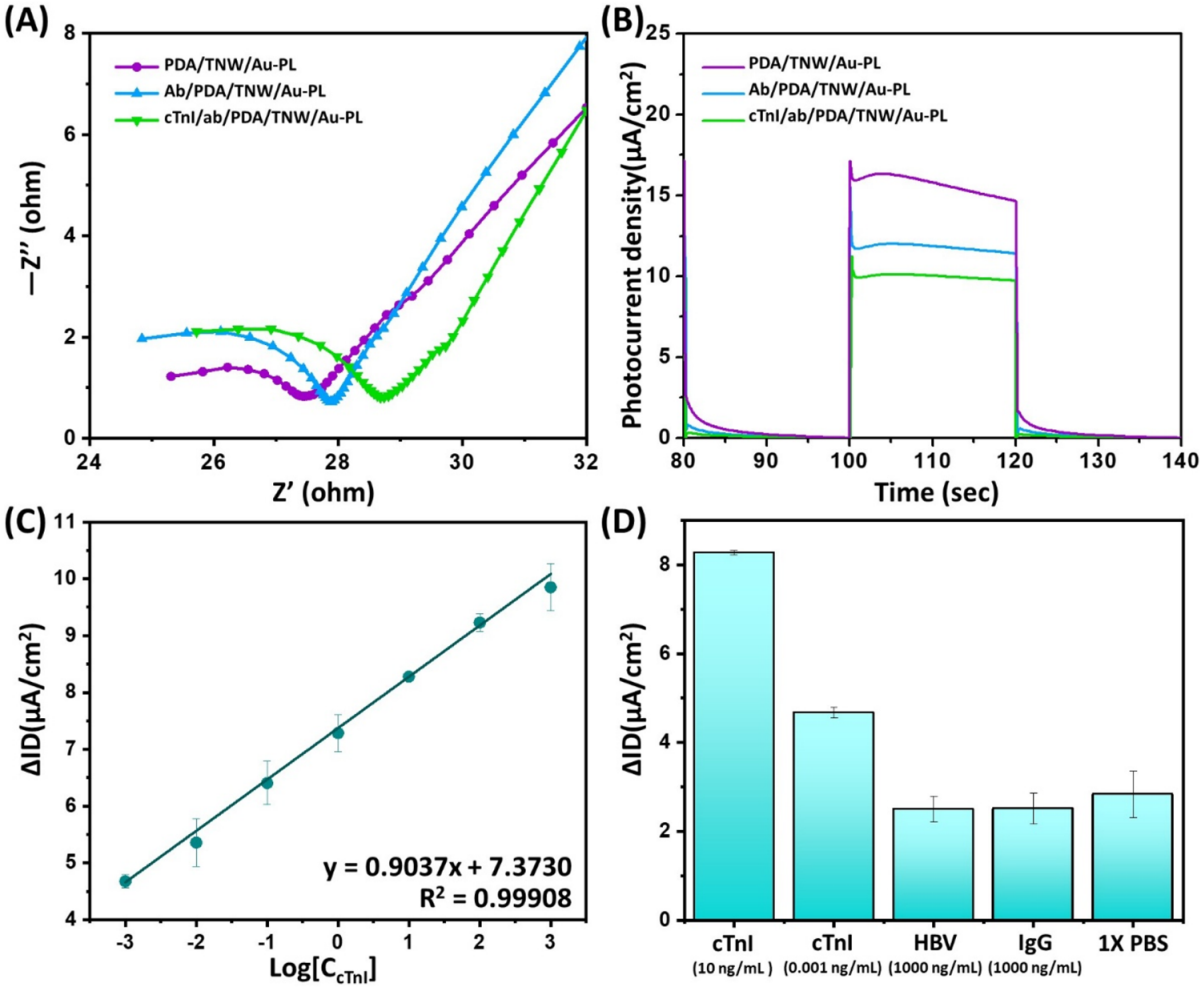
Detection of cTnI using a PDA/TNW/Au-PL-based photoelectrode. (A) EIS Nyquist plot of the PDA/TNW/Au-PL photoelectrode with and without bioconjugation of cTnI antibody and cTnI antigen, performed in a 0.5 mM [Fe­(CN)_6_]^3–^/^4–^ solution containing 0.1 M KCl in the frequency range of 0.01 and 1 MHz. (B) Photocurrent response of the proposed PDA/TNW/Au-PL, ab/PDA/TNW/Au-PL, and cTnI/ab/PDA/TNW/Au-PL. (C) Linear calibration curve for the detection of cTnI antigen in PBS. (D) Selectivity detection of the prepared photoelectric immunosensor for cTnI. The error bars represent the standard deviation calculated from the measurements taken across three independently fabricated photoelectrodes. For each data point, triplicate measurements under identical conditions were performed using separately designed immunosensors to capture the variability arising from the fabrication and measurement processes. The average values and corresponding standard deviations are then plotted to reflect reproducibility.

The developed immunosensor was then used to detect cTnI at different concentrations, following a 30 min cTnI incubation step prior to photocurrent measurement. The photocurrent density decreased with increasing concentration of cTnI, which is suitable for cTnI quantitative detection. A linear relationship between the photocurrent response and the logarithm of cTnI concentration was observed over the range of 0.001–1000 ng/mL. This behavior can be attributed to Langmuir-type binding kinetics, which are characteristic of antibody–antigen interactions on immobilized immunosensor surfaces.^61^ This model indicates saturation binding behavior, where the signal response would become gradually less sensitive at higher analyte concentrations due to the limited availability of binding sites. As a result, plotting the photocurrent response against the logarithm of the concentration effectively linearizes the sensor output over a wide dynamic range. Such a log-linear transformation is widely used in biosensor calibration to ensure accurate quantification over several orders of magnitude.^62,63^ In our system, the broad linear detection range from 0.001 to 1000 ng/mL is likely due to the high-density and stable immobilization of antibodies via the PDA layer coated on the porous TNW surface, which provides abundant binding sites with strong adhesion. Also, the enhanced light absorption and efficient charge separation facilitated by Au-PL significantly amplified the photoelectric signal, allowing sensitive detection even at trace levels of cTnI. The corresponding regression equation for the linear plot of the photocurrent response and the logarithm of cTnI concentration was Δ*I* = 0.90 log [*C*_cTnI_] + 7.38 (ng/mL), with a linear correlation coefficient of 0.999 (Figure 4C). The limit of detection was 0.001 ng/mL. Compared our results with other Au- or TiO_2_-based composite photoelectrochemical immunosensors, the sensing performance of our designed ab/PDA/TNW/Au-PL immunoassay is competitive to those in the literature, as listed in Table S1. Notably, there is still a gap to effectively utilize these reported sensors in PoCT devices for practical applications due to the complexity of the sensor device and high reagent consumption used for signal amplification, such as secondary antibodies, amplification labels, as well as redox mediators. However, our work provides a one-step and label-free photoelectric immunoassay to achieve excellent sensitivity and detection limits of cTnI-sensing photoelectrodes, which is more realistic and efficient than reported sensors due to its triple intensified functions for signal amplification arising from the synergistic integration of Au-PL, TNW, and PDA, with each component contributing distinctly to improved light harvesting, increased charge separation, and better biomolecular recognition. First, the formation of type II heterojunctions at the PDA/TNW interface and Schottky junctions at the TNW/Au-PL interface enables efficient charge separation and directional electron transport. The p-type semiconductor PDA and n-type semiconductor TiO_2_ would establish a type II band alignment that can promote efficient electron–hole pair separation by facilitating the transfer of photogenerated electrons from PDA to TiO_2_ while retaining holes within PDA. Simultaneously, the TiO_2_–Au interface forms a Schottky barrier that facilitates efficient electron extraction and prevents backflow, thereby further suppressing recombination losses and enhancing photocurrent generation. Second, strong absorption across the entire visible spectrum is achieved through the combined effects of the Au-PL and TNW nanostructures, which work together to enhance light harvesting by broadening and intensifying the light absorption across the visible spectrum. The Au-PL layer excites localized photonic modes and plasmonic resonances, thereby effectively extending the absorption range. Meanwhile, the TNW layer, with its pore-rich and cavity-rich antireflection structure and high specific surface area, can suppress surface reflectance and increase the optical path length by promoting scattering and multiple internal reflections, further boosting light trapping within the photoelectrode.^64^ This synergistic light management ensures that more incident photons are absorbed and further utilized for carrier generation, as evidenced by the suppressed reflectance and relatively low transmittance observed in optical measurements (Figures S4 and S5). Third, the PDA coated on the porous TNW surface can serve multiple roles, acting as a light-absorbing substance, an electron transfer improver by providing an energetically favorable interface to reduce the recombination of photogenerated photogenerated e^–^/h^+^, and a robust adhesive layer rich in functional groups for the effective immobilization of cTnI antibodies for further cTnI recognition (Figures S6 and S7). This multifunctionality not only improves light absorption and suppresses charge recombination but also enables effective biofunctionalization, which is beneficial for achieving high sensitivity and low detection limits in cTnI sensing.

The selectivity of the sensors is also significant for detecting cTnI. In order to evaluate the selectivity, interfering substances of Hepatitis B virus (HBV) and immunoglobulin G (IgG) are selected for testing. As shown in Figure 4D, no significant change in the photocurrent is observed for HBV and IgG in comparison with the results observed for cTnI, indicating that our designed photoelectric biosensor has good selectivity. All these results collectively demonstrate that the proposed PDA/TNW/Au-PL photoelectrode, featuring an inherent self-signal amplification property, is a good candidate for fabricating cTnI sensors with satisfactory selectivity and sensitivity. To preliminarily verify the sensing performance of our platform in biological samples, we performed a comparative test using a blood glucose-like test strip format.^24^ The photocurrent change (ΔID) was measured for cTnI at 0.001 and 10 ng/mL in both PBS and diluted human serum. As shown in Figure S8, the sensor displayed a clear concentration-dependent increase in the signal in both matrices. Notably, the signal intensity in serum is comparable to that in PBS, indicating that the sensor maintains its sensitivity even in complex biological fluids. These results not only demonstrate our platform’s compatibility with serum but also highlight its potential for integration into point-of-care diagnostic formats. In alignment with the goal of practical PoCT applications, further work to validate the performance in clinical settings will be conducted under Taiwan IRB regulations. Moreover, the estimated manufacturing cost per sensing device is low, which is approximately USD 2.65–3.15. The sensing platform also displays the potential for batch fabrication due to its quite simple and straightforward fabrication process. We believe that the combination of cost efficiency and strong sensing performance highlights the platform’s commercialization potential, particularly for point-of-care diagnostics in the detection of low-abundance small biomolecules.

## 3 Conclusions

In summary, we developed a sensitive and one-step assembled photoelectric immunosensor based on a hybrid trilayer plasmonic photoelectrode of PDA/TWN/Au-PL. The significant aspects of this development lie in its triple-function signal intensification characteristics arising from the synergistic integration of Au-PL, TNW, and PDA for enhancing the sensing performance, including (1) interfacial layer engineering, where a type II heterojunction is formed at the PDA/TNW interface and a Schottky junction at the TNW/Au-PL interface, for facilitating effective charge separation and good electron transfer capability, (2) 2D Au plasmonic layer combined with porosity-rich and cavity-rich TiO_2_ nanowires for providing strong absorbance in the whole visible light range, and (3) PDA as the heterojunction-promoting functional coating multiple for improving photoactive performance and also as the adhesive interlayer for increasing antibody immobilization capacity. With the above signal amplification advantages, good analytical performance for one-step label-free cTnI detection can be obtained under homemade white LED illumination. It shows competitive sensitivity for cTnI, with a wide range of 0.001–1000 ng/mL and a detection limit of 0.001 ng/mL, making it applicable for sample analysis in typical clinical settings. The selectivity is also confirmed through interference tests. Although the long-term stability of the PDA layer under continuous illumination and standard validation procedures, such as recovery testing and direct comparison with clinical reference methods like the enzyme-linked immunosorbent assay (ELISA), could not be completed within the scope of this study, the above results can still support the analytical reliability of our developed photoelectric immunosensing platform. In addition to the sensitivity and selectivity, the overall performance of the photoelectric immunosensor along with its short detection time for one-step detection of biotargets, low cost, and potential batch fabrication is particularly advantageous for practical applications such as the integration with portable and smartphone-based detection systems.^65,66^ Most importantly, the obtained characteristics are particularly advantageous for detecting small biomolecules or low analyte count clinical scenarios, without any need for labels and amplifiers, positioning it as a highly promising device for practical PoCT applications.

## 4 Experimental Section

### 4.1 Fabrication of the PDA/TNW/Au-PL Photoelectrode

The layer-by-layer PDA/TNW/Au-PL was prepared in three steps, as shown in Figure S2. First, the SPR bottom layer of the Au-PL surface was deposited onto the FTO surface. The plasmonic surface of a densely random-packed Au-nanoparticle layer with a wide size was formed by sputtering deposition, followed by microwave plasmon heat treatment, in which a 3 ∼ 6 nm gold film was sputter-deposited onto the FTO substrate and then created isolated Au nanoparticles through thermal annealing treatment under microwave conditions. This procedure was repeated six times to achieve a well-defined 2D array of Au-patterned layers. Second, high-porosity TiO_2_ nanowires were grown on the Au-PL layer to form a plasmonic hybrid bilayer (TNW/Au-PL). The fabrication process of TNW is performed by initially depositing a 50 nm-thick Ti film onto the Au-PL surface using the magnetic sputtering method and subsequently transferring Ti into cavity-rich nesting TiO_2_ nanowire layers by a simple alkaline thermal treatment in 2.5 M NaOH aqueous solution at 80 °C for 1 h. After washing with nitric acid and ethanol and annealing at 500 °C for 1 h, the TNW/Au-PL plasmonic hybrid bilayer was obtained. Finally, a PDA coating was applied on TNW/Au-PL to form a trilayer PDA/TNW/Au-PL photoelectrode. Before the PDA self-assembly process, the TNW/Au-PL surface was treated with the plasma for 10 min to clean and activate it to improve the adhesion between the surface and solution. After soaking TNW/Au-PL in a 2 mg/mL dopamine solution, which was preprepared by the addition of 2 mg dopamine in 1 mL of tris-buffer (pH 8.4) for 1.5 h at 4 °C, the final photoelectrode of PDA/TNW/Au-PL was obtained after washing with Milli-Q water and drying with a nitrogen flow gas.

### 4.2 Construction of the PDA/TNW/Au-PL-Based Photoelectric Immunosensing Platform: ab/PDA/TNW/Au-PL

Photoelectric performance was verified by an immunosensing platform, which was designed with three electrodes composed of the as-prepared hybrid layer-by-layer photoelectrode as the working electrode, conducting carbon as the counter electrode, and Ag/AgCl as the reference electrode. The anti-cTnI antibody (Hytest, 8RTI7) was first diluted to 1000 ng/mL in 1× phosphate-buffered saline (PBS) and stored at −4 °C. Subsequently, the PDA/TNW/Au-PL photoelectrode was incubated with 95 μg/mL cTnI antibody for 10–40 min to determine the ideal incubation time. The photocurrent change increased with increasing incubation time and reached an optimized incubation time of 10 min (Figure S6A). After 10 min of incubation with anti-cTnI, the assembled photoelectric immunosensor (denoted as ab/PDA/TNW/Au-PL) was used for cTnI detection after rinsing with Milli-Q water, followed by drying at ambient temperature under a nitrogen flow. Therefore, we know that our designed PDA/TNW/Au-PL immunosensing platform has the potential for batch fabrication due to its simple, scalable, and modular fabrication process, involving well-established techniques commonly used in nanomaterial and biosensor manufacturing, which is essential for commercialization and large-scale deployment in clinical diagnostics.

## 4.3 Characterization

Element mapping, cross-sectional morphology, and structure of the prepared samples were investigated using high-resolution transmission electron microscopy (HRTEM, JEM 2010, 200 kV). Field emission scanning electron microscopy (FESEM, Zeiss Ultra Plus) with an accelerating voltage of 3 kV was used for the characterization of surface morphology and chemical composition of the samples. The formation of PDA on TNW/Au-PL was analyzed using Fourier-transform infrared (FTIR) spectroscopy, which was conducted using a PerkinElmer Spectrum 100. UV–vis absorption spectra were obtained using a UV–vis–NIR spectrophotometer (SHIMADZU UV-3600 Plus) with BaSO_4_ as a reference. The absorbance (*A*) of the samples can be obtained using the equation *A* = 100% – *T* – *R*, where *T* is the transmittance and *R* is the reflectance.

## 4.4 Detection of cTnI

Photoelectric performance was evaluated using a three-electrode system composed of the as-prepared ab/PDA/TNW/Au-PL as the working electrode, conducting carbon as the counter electrode, and Ag/AgCl as the reference electrode in 90 mL of PBS solution at an applied potential of 0.6 V under homemade white LED illumination. This homemade white LED light source was used for all the photocurrent measurements. The light source was constructed using an XHP70.2 1A-N4 LED lamp bead operating at 30 W with a luminous flux of 1800 lm. The LED emits broadband white light across the visible spectrum (400–7780 nm), providing sufficient and consistent illumination for photoelectrochemical experiments.

First, the cTnI stock solution (Hytest, 19C7) was dispersed in 100 times diluted Human Serum (Merck Millipore, S1 – 100 mL) at different concentrations (0.001, 0.01, 0.05, 0.1, 1, 10, 50, 100, and 1000 ng/mL). Subsequently, the cTnI solution is dropped onto the working electrode for detection with an optimal incubation time of 30 min at room temperature (Figure S6B). After washing with Human Serum dilution and drying with nitrogen, the samples were analyzed using the photoelectric immunosensor developed in this work by using an electrochemical workstation (CHI661E, CH Instruments). The sensing area was defined as around 0.7 cm^2^. The sensing performance is evaluated by monitoring the changes in photocurrent, which is calculated as the average current over the entire 20 s aperture time (light-on period) to ensure signal stability and reduce the influence of transient fluctuations.

## Supplementary Material



## Notes

Fourier-transform infrared spectra, transmittance spectra, reflectance spectra, and characterization of PDA/TNW/Au-PL, TNW/Au-PL, PDA/Au-PL, and Au-PL properties; schematic representation of the proposed one-step photoelectric immunosensing platform for the direct biomarker detection; optimization of the incubation time of cTnI antibody and antigen; and preliminary evaluation of the sensing performance of our designed sensor in diluted human serum (PDF)

## Abbreviations

AMI: acute myocardial infarction
WHO: World Health Organization
cTnI: cardiac troponin I
ECL: electrochemiluminescence
ELISA: enzyme-linked immunosorbent assay
PoCT: point-of-care testing
2D: two-dimensional
AFP: α-fetoprotein
IgG: immunoglobulin G
PDA: polydopamine
FTIR: Fourier-transform infrared
EDS: energy-dispersive spectrometry
HOMO: highest occupied molecular orbital
LUMO: lowest occupied molecular orbital
SPR: surface plasmon resonance
EIS: electrochemical impedance spectroscopy
HBV: hepatitis B virus
