# Supplementary material for: Triple-Function-Intensified Photoelectric Immunosensing Platform for One-Step Detection of Cardiac Troponin I
Source: ACS Appl Mater Interfaces. 2025 Aug 21;17(35):49965–75. doi: 10.1021/acsami.5c06443 (PMC12412093; doi:10.1021/acsami.5c06443)
Supplement: Supplementary file 1 [file am5c06443_si_001.pdf]

Supporting Information

Triple-Function Intensified Photoelectric Immunosensing Platform for One-step Detection of Cardiac Troponin I

Shin-Chwen Yeh, Wen-Shan Chen, Wen-Yin Ko,* Kuan-Jiuh Lin*

Department of Chemistry, National Chung Hsing University, Taichung, 402, Taiwan.
E-mail: kjlin@nchu.edu.tw; wyko@nchu.edu.tw

Table S1. Photoelectrochemical biosensor based on Au- or TiO_2- materials for the detection of cardiac troponin I.

**Sample**	One-step incubation and detection	Block site	Light	Antibody incubation time (min)	Detection time (min)	Detection limit (ng/mL)	Linear range (ng/mL)	**Ref.**
InGaZnO (IGZO) /Cr/Au	X	O	LED lamp	60	35	1.5×10^{-2}	0.15–50	1
NAC-CdAgTe QDs/AuNPs/GCE	X	O	100 W LED lamp	60	65	1.756×10^{-3}	0.005–0.02	2
ITO/FeOOH/Bi_2S_3/Au	X	O	100 W LED lamp	30	-------	7.6×10^{-4}	0.001–100	3
PSATs/CdS	X	X	500 W Xe lamp	-------	240	4.7×10^{-4}	0.0012–20	4
Ag@Cu_2O core-shell SPs/TiO_2/CdS	X	O	LED lamp	30	60	6.7×10^{-6}	0.00002–50	5
CdSe/CdS/ZnS@TiO_2	X	O	UV lamp	420	30	1.442×10^{-2}	0.01–0.2	6
Au/MWCNTs nanohybrids on N-GQDs/CdS QDs dual sensitized urchin-like TiO_2	X	O	100 W LED lamp	60	60	6.16×10^{-6}	0.0001–50	7
ITO/ZTCHS/$Ru(bpy)_3^{2+}$/Bi_2S_3	X	O	100 W LED lamp	-------	-------	3×10^{-5}	0.0001–100	8
CM-dextran/Au/TiO_2 NTA/Ti	X	X	500 W Xe lamp	60	60	2.2×10^{-2}	0.0484–484	9
Nanoporous CdS QDs/TiO_2 NTs	X	O	500 W Xe lamp	60	240	5×10^{-4}	0.0005–10	10
**PDA/TNW/Au-PL**	O	X	29 W LED lamp	**10**	**31**	1×10^{-3}	0.001–1000	**This work**

Table S2. Comparison of detection performances for cardiac troponin I using Au- materials between the developed photoelectric sensing platform and other strategies.

Strategy	Advantages	Disadvantages	Material	Antibody incubation time (min)	Detection time (min)	Detection limit (ng/mL)	Linear range (ng/mL)	Ref.
**Colorimetric**	· Cost-effective, rapid, and easy to operate<br>· Requires only a small sample volume<br>· Applicable to both vapor and liquid-phase analysis	· Limited reproducibility<br>· Short shelf-life and stability<br>· Generates large RGB datasets that require chemometric analysis	PDMS–Au-Ag	720	90	1×10^{-2}	0.01–10	11
			PDMS–Au-Ag	60	60	5×10^{-1}	0.5–50	12
			Au@Ag–Pt	20	70	2×10^{-2}	0.02–1	13
			Au@Pt	720	97	3.4×10^{-1}	0.5–5	14
			PBDT@Au NPs	30	15	3×10^{-2}	0.06–500	15
**Fluorescence**	· Reliable, cost-effective, and rapid detection platform<br>· Easy operation with fast response time<br>· Simple instrumentation	· Require bright fluorescent tags and signal amplification strategies<br>· Limited photostability<br>· Susceptible to environmental interferences	ORLA85 /PEG-thiol/ Au/Ti	Overnight	60	9.8×10^{-1}	3.9–100	16
			ZIF-8 @BSA Au /AgNCs	60	10	9×10^{-2}	0.01–0.15	17
			FMGC	120	40	1×10^{-1}	0.1–100	18
			AuNC @GSH	45	5	9.1×10^{-1}	5–45	19
**SPR**	· In situ assessment, inexpensive, and simple to develop<br>· Enables rapid, unlabeled proteins detection<br>· Low-cost optical setup	· Sensitivity is highly dependent on the distance between the analyte and the sensor surface<br>· Poor response to small molecules or weak refractive index changes<br>· Challenge to use in trace detection	Au film Dextran layer	20	35	1.4	1–3	20
			Au thin film	12-15	28	3.12×10^{-2}	0.03125–0.25	21
			Au film-PDA-AuNPs	180	70	3.75	15–2500	22
**Photoelectro-chemical**	· Binder-free sensor: without amplification strategies such as the usage of labeling materials, blocking reagents and linker molecules<br>· Enables ultrasensitive detection with a high signal-to-noise ratio<br>· Effective for small molecule analysis<br>· Low background interference<br>· Simple, efficient, and cost-effective	· Insufficient long-term stability of the PDA layer under illumination	**PDA /TNW /Au-PL**	**10**	**31**	**1×10^{-3}**	**0.001–1000**	**This work**

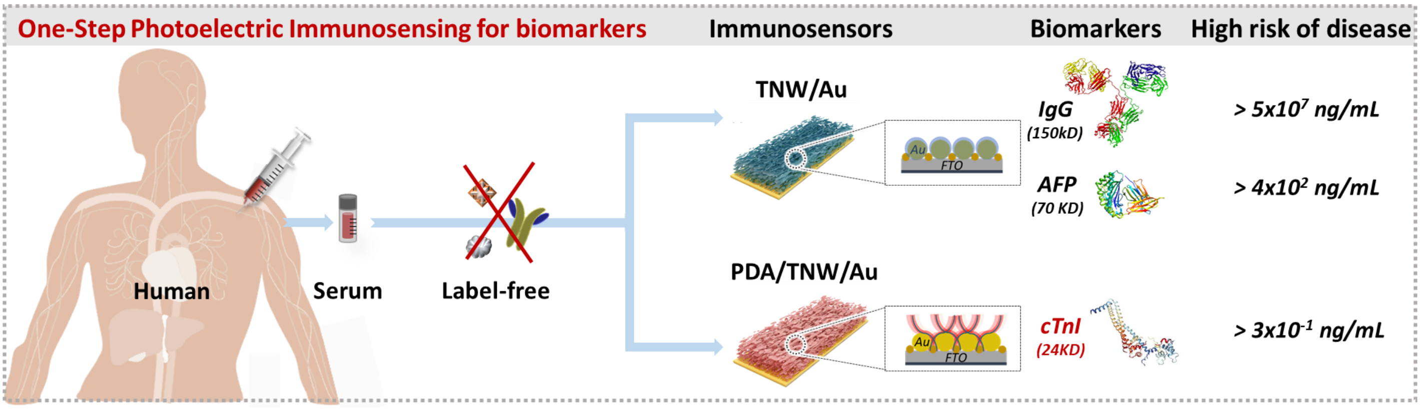

Figure S1. Schematic representation of proposed one-step photoelectric immunosensing platform for the direct biomarker detection under home-made visible light. This protocol eliminates the need of linkers, blocking agents, and secondary antibodies, streamlining the process while maintaining high sensitivity.

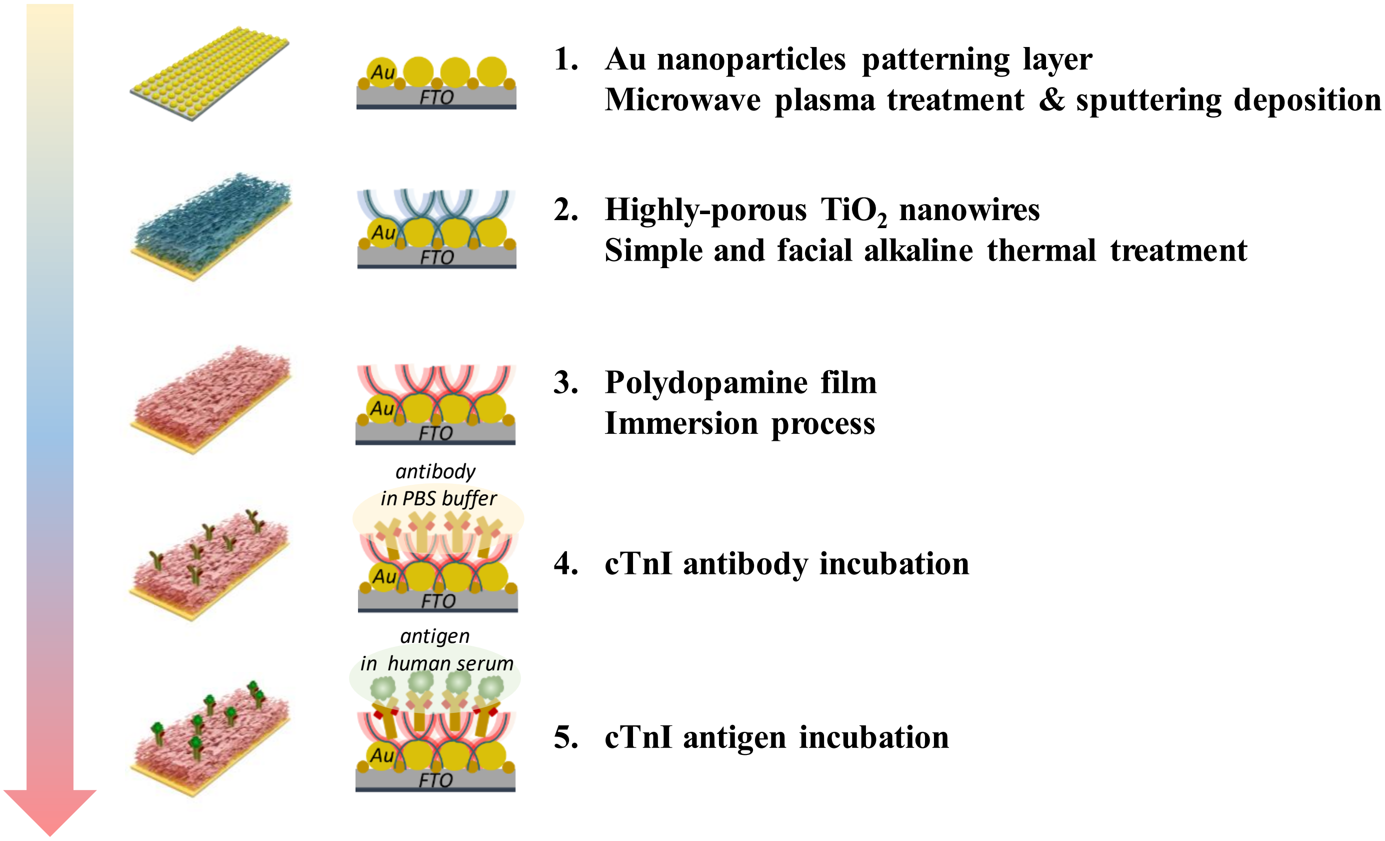

Figure S2. Schematic diagram of the fabrication process of ab/PDA/TNW/Au-PL photoelectric immunosensor for the detection of cTnI.

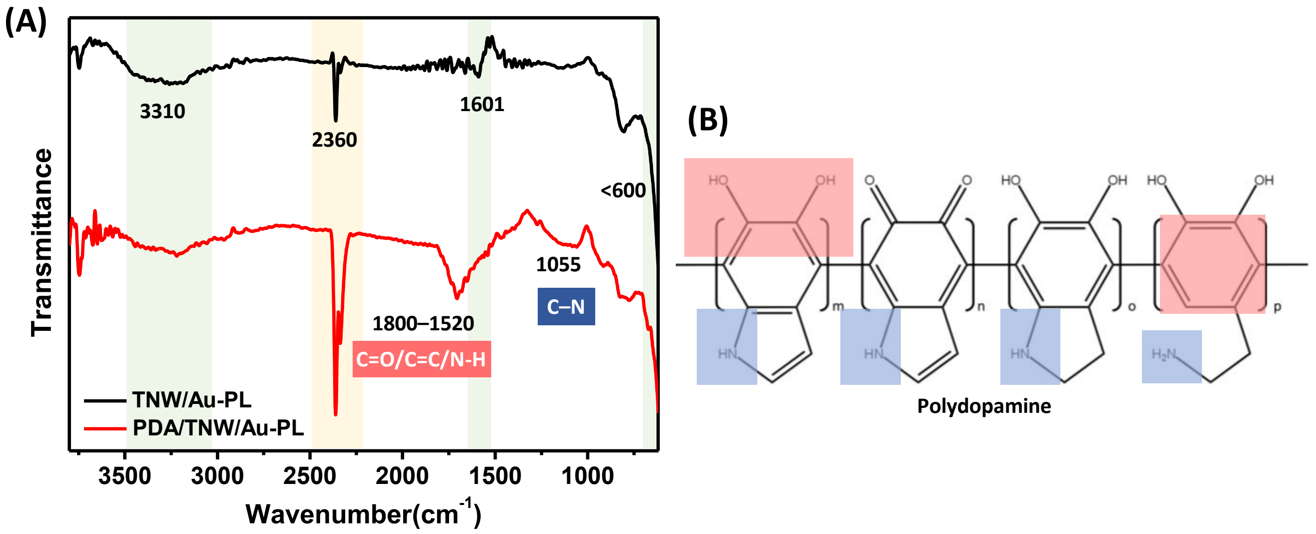

Figure S3. (A) Fourier transform infrared (FTIR) of PDA/TNW/Au-PL and TNW/Au-PL. (B) Structural diagram of PDA.

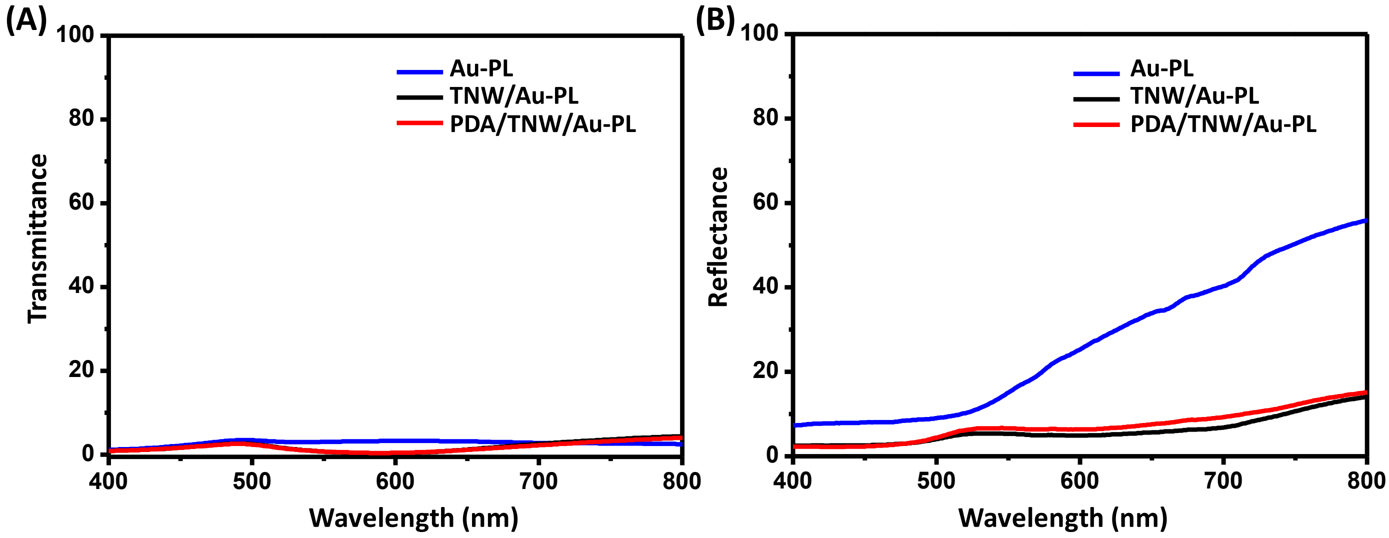

Figure S4. (A) Transmittance spectra and (B) Reflectance spectra of Au-PL, TNW/Au-PL, and PDA/TNW/Au-PL.

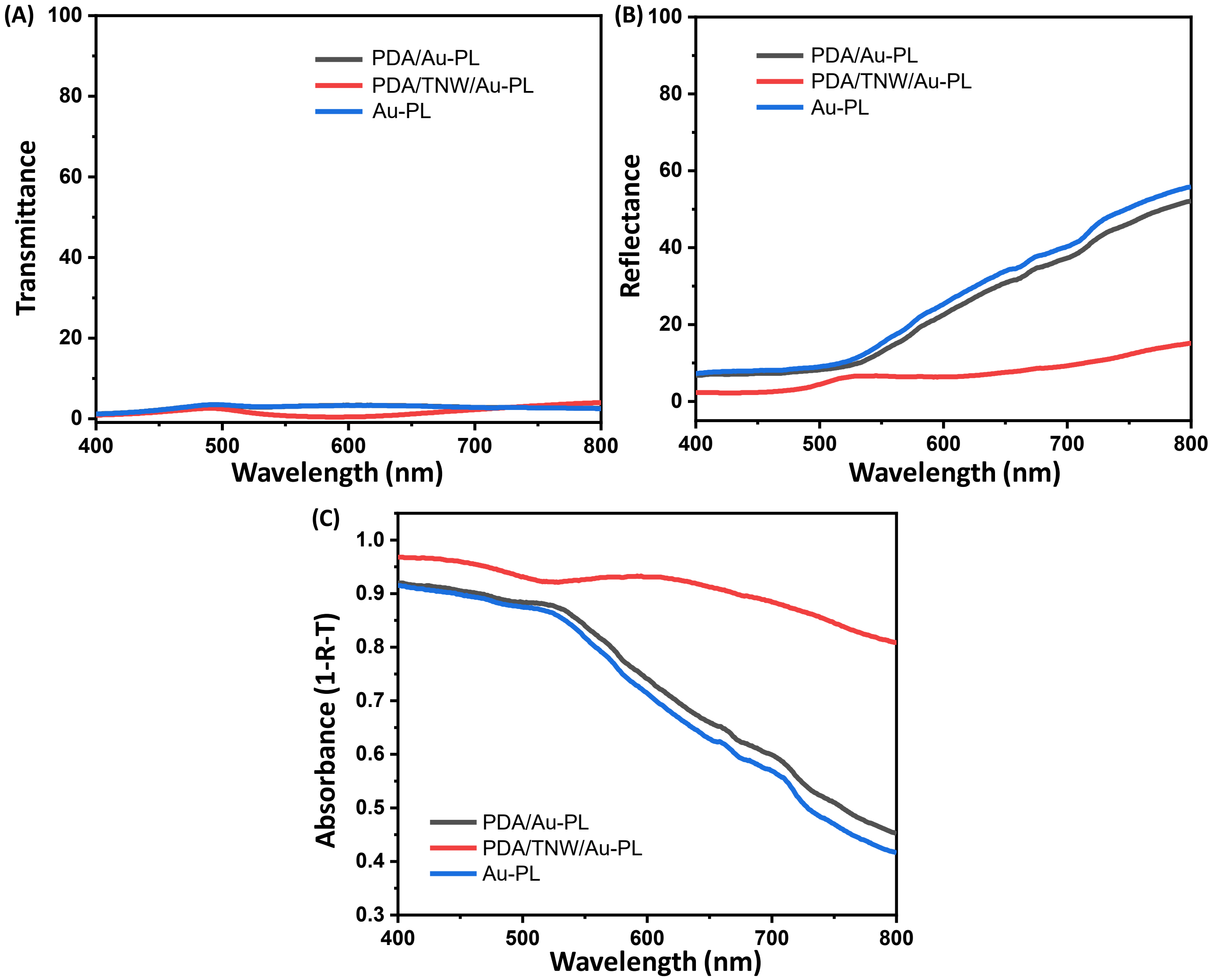

Figure S5. (A) Transmittance spectra, (B) Reflectance spectra, and (C) Absorbance spectra of the control photoelectrodes compared to the designed PDA/TNW/Au-PL photoelectrode. From the transmittance data, we could observe that all photoelectrodes exhibit very low transmittance across the visible range (400-800 nm). From the reflectance data, Au-PL and PDA/Au-PL photoelectrodes presenting relatively high reflectance are obtained, implying that a portion of incident light is reflected and not utilized for carrier generation. In contrast, the PDA/TNW/Au-PL exhibits markedly reduced reflectance in the range of 400-800 nm, indicating that more incident light is effectively trapped and absorbed within this photoelectrode. The results clearly indicates that the TNW layer can serve as an anti-reflective coating for enhancing light trapping capability by effectively suppressing the reflectance loss, owing to its pore-rich and cavity-rich framework that can scatter and redirect incident light, increase optical path length within the absorbing layers, and promote multiple internal reflections.

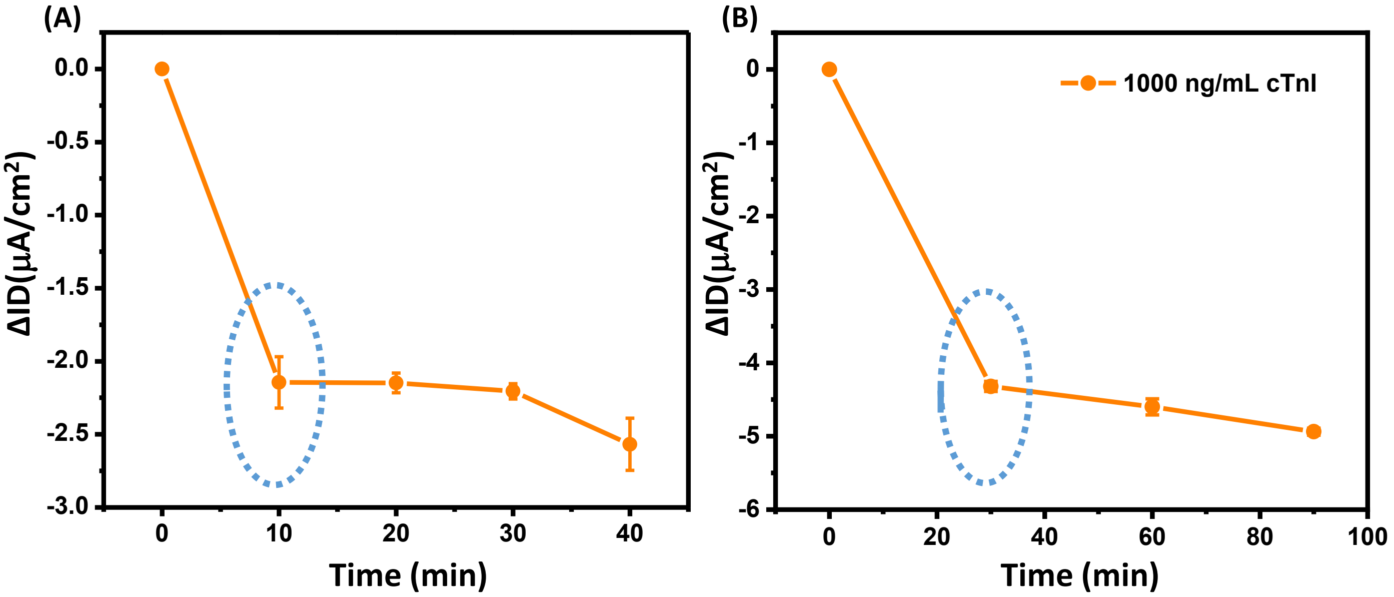

Figure S6. Optimization of the incubation time of (A) cTnI antibody and (B) cTnI antigen by using the PDA/TNW/Au-PL photoelectrode for photoelectric immunosensing.

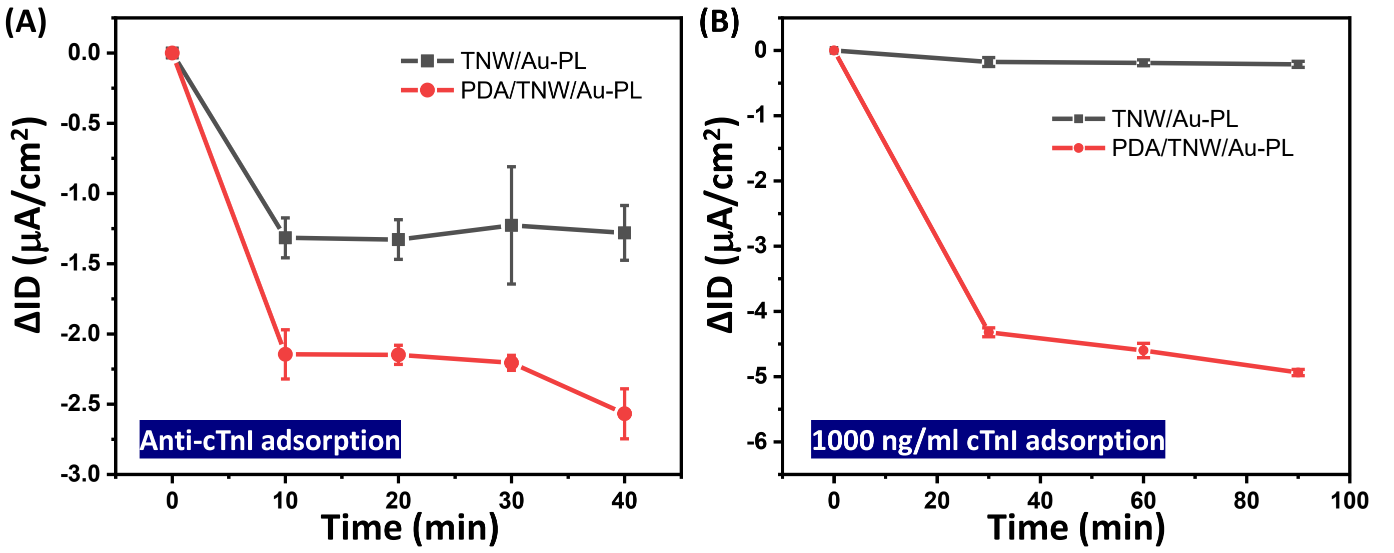

Figure S7. Photocurrent change as a function of incubation time in (A) cTnI antibody and (B) cTnI antigen solutions by using PDA/TNW/Au-PL and TNW/Au-PL without PDA photoelectrodes for photoelectric immunosensing. From the data, we could observe that the PDA/TNW/Au-PL photoelectrode exhibits a significantly greater photocurrent change (ΔID) after anti-cTnI incubation as compared to the TNW/Au-PL one, indicating more efficient anti-cTnI immobilization. This can be attributed to PDA's high surface area and abundant catechol and amine groups that enable effective and strong binding on to the TNW surface. After 10-minute anti-cTnI incubation, the immunosensing sensor is then exposed to 1000 ng/mL cTnI, where the PDA/TNW/Au-PL again shows a larger ΔID, confirming more effective antigen capture. In contrast, the TNW/Au-PL one shows much weaker photocurrent change signals, likely due to limited antibody loading and weak physical adsorption that may cause antibody desorption or denaturation during cTnI incubation process, ultimately resulting in ineffective cTnI recognition and compromising sensing performance.

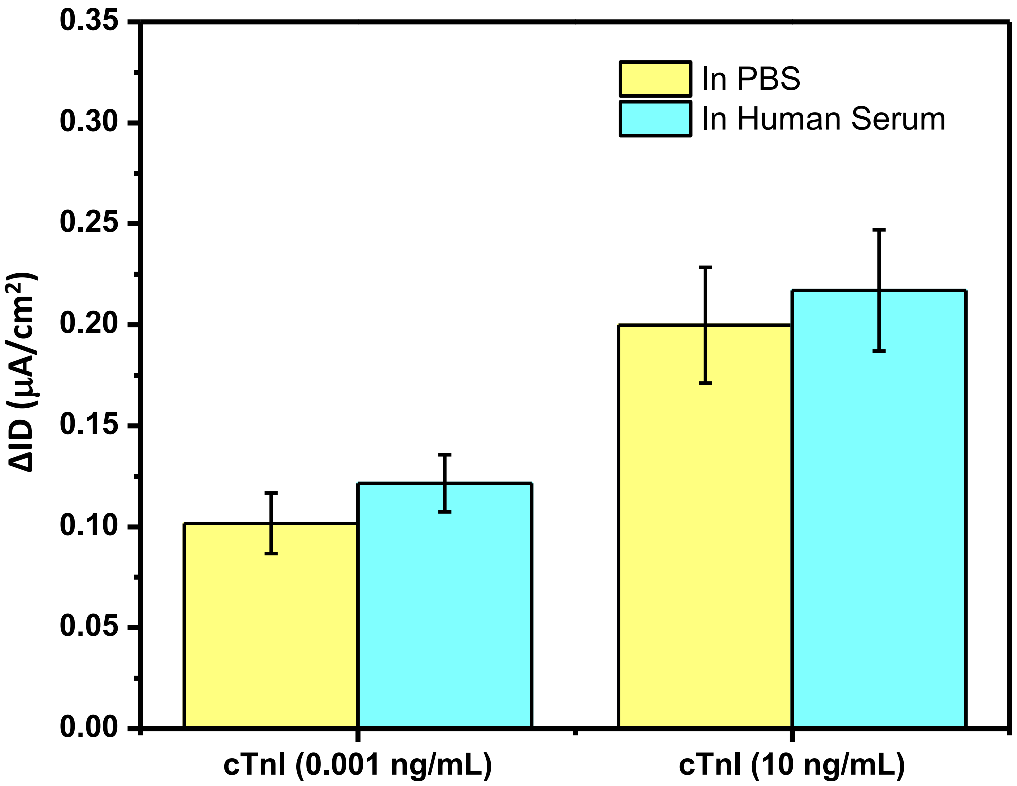

Figure S8. Photocurrent change (ΔID) of our proposed sensing platform toward cTnI at 0.001 and 10 ng/mL in PBS and 100 times diluted human serum, measured using a blood glucose-like test strip format with a sensing area of 0.15 cm^2 at an applied potential of 0.4 V under home-made white LED illumination. The platform exhibits a clear concentration-dependent signal increase in both matrices, with comparable responses in serum and PBS, demonstrating its feasibility for detection in complex biological fluids and its potential for point-of-care diagnostic applications.

References:

1 Han, X., Shokri Kojori, H., Leblanc, R. M. & Kim, S. J. Ultrasensitive Plasmonic Biosensors for Real-Time Parallel Detection of Alpha-L-Fucosidase and Cardiac-Troponin-I in Whole Human Blood. *Analytical Chemistry* **90**, 7795-7799, doi:10.1021/acs.analchem.8b01816 (2018).

2 Tan, Y. *et al.* Enhanced photoelectrochemical immunosensing of cardiac troponin I based on energy transfer between N-acetyl-L-cysteine capped CdAgTe quantum dots and dodecahedral Au nanoparticles. *Biosensors and Bioelectronics* **91**, 741-746, doi:https://doi.org/10.1016/j.bios.2017.01.040 (2017).

3 Bao, C. *et al.* Cardiac troponin I photoelectrochemical sensor: {Mo368} as electrode donor for Bi2S3 and Au co-sensitized FeOOH composite. *Biosensors and Bioelectronics* **157**, 112157, doi:https://doi.org/10.1016/j.bios.2020.112157 (2020).

4 Gao, C. *et al.* Paper based modification-free photoelectrochemical sensing platform with single-crystalline aloe like TiO2 as electron transporting material for cTnI detection. *Biosensors and Bioelectronics* **131**, 17-23, doi:https://doi.org/10.1016/j.bios.2019.01.038 (2019).

5 Chen, J. *et al.* Ultrasensitive photoelectrochemical immunosensor of cardiac troponin I detection based

on dual inhibition effect of Ag@Cu2O core-shell submicron-particles on CdS QDs sensitized TiO2 nanosheets. *Biosensors and Bioelectronics* **117**, 340-346, doi:https://doi.org/10.1016/j.bios.2018.05.037 (2018).

6 Memon, R., Shaheen, I., Qureshi, A. & Niazi, J. H. Enhanced detection of cardiac troponin-I using CdSe/CdS/ZnS core-shell quantum dot/TiO2 heterostructure photoelectrochemical sensor. *Journal of Alloys and Compounds* **1008**, 176592, doi:https://doi.org/10.1016/j.jallcom.2024.176592 (2024).

7 Sun, X. *et al.* A novel ultrasensitive sandwich-type photoelectrochemical immunoassay for PSA detection based on dual inhibition effect of Au/MWCNTs nanohybrids on N-GQDs/CdS QDs dual sensitized urchin-like TiO2. *Electrochimica Acta* **333**, 135480, doi:https://doi.org/10.1016/j.electacta.2019.135480 (2020).

8 Bao, C. *et al.* A signal-off type photoelectrochemical immunosensor for the ultrasensitive detection of procalcitonin: Ru(bpy)32+ and Bi2S3 co-sensitized ZnTiO3/TiO2 polyhedra as matrix and dual inhibition by SiO2/PDA-Au. *Biosensors and Bioelectronics* **142**, 111513, doi:https://doi.org/10.1016/j.bios.2019.111513 (2019).

9 Guo, W., Wang, J., Guo, W., Kang, Q. & Zhou, F. Interference-free photoelectrochemical immunoassays using carboxymethylated dextran-coated and gold-modified TiO2 nanotube arrays. *Analytical and Bioanalytical Chemistry* **413**, 4847-4854, doi:10.1007/s00216-021-03442-0 (2021).

10 Xue, T.-Y. *et al.* Nanoporous Semiconductor Electrode Captures the Quantum Dots: Toward Ultrasensitive Signal-On Liposomal Photoelectrochemical Immunoassay. *Analytical Chemistry* **91**, 3795-3799, doi:10.1021/acs.analchem.9b00170 (2019).

11 Wu, W.-Y., Bian, Z.-P., Wang, W., Wang, W. & Zhu, J.-J. PDMS gold nanoparticle composite film-based silver enhanced colorimetric detection of cardiac troponin I. *Sensors and Actuators B: Chemical* **147**, 298-303, doi:https://doi.org/10.1016/j.snb.2010.03.027 (2010).

12 Poosinuntakul, N., Chanmee, T., Porntadavity, S., Chailapakul, O. & Apilux, A. Silver-enhanced colloidal gold dip strip immunoassay integrated with smartphone-based colorimetry for sensitive detection of cardiac marker troponin I. *Scientific Reports* **12**, 19866, doi:10.1038/s41598-022-24458-1 (2022).

13 Bai, T. *et al.* Strategic synthesis of trimetallic Au@Ag–Pt nanorattles for ultrasensitive colorimetric detection in lateral flow immunoassay. *Biosensors and Bioelectronics* **208**, 114218, doi:https://doi.org/10.1016/j.bios.2022.114218 (2022).

14 Jiao, L. *et al.* Au@Pt nanodendrites enhanced multimodal enzyme-linked immunosorbent assay. *Nanoscale* **11**, 8798-8802, doi:10.1039/C8NR08741E (2019).

15 Yao, Y., Li, Y., Hua, Q., Zhao, W. & Li, J. Development of novel poly(1,4-benzenedithiol) nanoparticles@AuNPs lateral flow immunochromatographic test strips. *Analytical Methods* **17**, 2997-3006, doi:10.1039/D5AY00346F (2025).

16 Toma, K., Oishi, K., Iitani, K., Arakawa, T. & Mitsubayashi, K. Surface plasmon-enhanced fluorescence immunosensor for monitoring cardiac troponin I. *Sensors and Actuators B: Chemical* **368**, 132132, doi:https://doi.org/10.1016/j.snb.2022.132132 (2022).

17 Mirzaeizadeh, Z., Amin Sadrabadi, E., Naseri, N., Golmohammadi, H. & Omidfar, K. Smart early diagnosis of acute myocardial infarction: a ZIF-based nanofluorescence lateral flow immunoassay for

point-of-care detection of cTnI. *Materials Advances* **6**, 839-848, doi:10.1039/D4MA01000K (2025).

18 Song, S. Y., Han, Y. D., Kim, K., Yang, S. S. & Yoon, H. C. A fluoro-microbead guiding chip for simple and quantifiable immunoassay of cardiac troponin I (cTnI). *Biosensors and Bioelectronics* **26**, 3818-3824, doi:https://doi.org/10.1016/j.bios.2011.02.036 (2011).

19 Madanan, A. S. *et al.* Fluorescence anisotropic probe for sensing cardiac troponin-I antigen through target-specific antibody-conjugated gold nanoclusters. *Analytical Methods* **16**, 6899-6906, doi:10.1039/D4AY01240B (2024).

20 Masson, J. F., Obando, L., Beaudoin, S. & Booksh, K. Sensitive and real-time fiber-optic-based surface plasmon resonance sensors for myoglobin and cardiac troponin I. *Talanta* **62**, 865-870, doi:https://doi.org/10.1016/j.talanta.2003.09.032 (2004).

21 Sinha, R. K. Wavelength modulation based surface plasmon resonance sensor for detection of cardiac marker proteins troponin I and troponin T. *Sensors and Actuators A: Physical* **332**, 113104, doi:https://doi.org/10.1016/j.sna.2021.113104 (2021).

22 Chen, F. *et al.* Fe3O4@PDA immune probe-based signal amplification in surface plasmon resonance (SPR) biosensing of human cardiac troponin I. *Colloids and Surfaces B: Biointerfaces* **177**, 105-111, doi:https://doi.org/10.1016/j.colsurfb.2019.01.053 (2019).
